# Disruption of *PHO13* improves ethanol production via the xylose isomerase pathway

**DOI:** 10.1186/s13568-015-0175-7

**Published:** 2016-01-14

**Authors:** Takahiro Bamba, Tomohisa Hasunuma, Akihiko Kondo

**Affiliations:** Department of Chemical Science and Engineering, Graduate School of Engineering, Kobe University, 1-1 Rokkodai, Nada, Kobe, 657-8501 Japan; Organization of Advanced Science and Technology, Kobe University, 1-1 Rokkodai, Nada, Kobe, 657-8501 Japan; Biomass Engineering Program, RIKEN, 1-7-22 Suehiro-cho, Tsurumi-ku, Yokohama, Kanagawa 230-0045 Japan

**Keywords:** Bioethanol, PHO13, *Saccharomyces cerevisiae*, Xylose fermentation, Xylose isomerase

## Abstract

Xylose is the second most abundant sugar in lignocellulosic materials and can be converted to ethanol by recombinant *Saccharomyces cerevisiae* yeast strains expressing heterologous genes involved in xylose assimilation pathways. Recent research demonstrated that disruption of the alkaline phosphatase gene, *PHO13*, enhances ethanol production from xylose by a strain expressing the xylose reductase (XR) and xylitol dehydrogenase (XDH) genes; however, the yield of ethanol is poor. In this study, *PHO13* was disrupted in a recombinant strain harboring multiple copies of the xylose isomerase (XI) gene derived from *Orpinomyces* sp., coupled with overexpression of the endogenous xylulokinase (XK) gene and disruption of *GRE3*, which encodes aldose reductase. The resulting YΔGP/XK/XI strain consumed 2.08 g/L/h of xylose and produced 0.88 g/L/h of volumetric ethanol, for an 86.8 % theoretical ethanol yield, and only YΔGP/XK/XI demonstrated increase in cell concentration. Transcriptome analysis indicated that expression of genes involved in the pentose phosphate pathway (*GND1*, *SOL3*, *TAL1*, *RKI1*, and *TKL1*) and TCA cycle and respiratory chain (*NDE1*, *ACO1*, *ACO2*, *SDH2*, *IDH1*, *IDH2*, *ATP7*, *ATP19*, *SDH4*, *SDH3*, *CMC2*, and *ATP15*) was upregulated in the YΔGP/XK/XI strain. And the expression levels of 125 cell cycle genes were changed by deletion of *PHO13*.

**Electronic supplementary material:**

The online version of this article (doi:10.1186/s13568-015-0175-7) contains supplementary material, which is available to authorized users.

## Introduction

Bioethanol is widely viewed as a potential new energy source and alternative to fossil fuels. Lignocellulosic materials, which represents an abundant, inexpensive, and renewable resource, are of great interest as a feedstock for bioethanol production. *Saccharomyces cerevisiae* is an ideal host for bioethanol production due to its high stress tolerance, high ethanol production capacity, and ease of gene manipulation. However, *S. cerevisiae* cannot inherently assimilate xylose, the second most abundant sugar in lignocellulosic biomass (Mosier et al. 2005).

Xylose-assimilating yeast strains have been constructed through genetic engineering to exploit two different heterologous xylose-utilization pathways. The oxidoreductase pathway involves the conversion of xylose to xylitol by xylose reductase (XR) and the conversion of xylitol to xylulose by xylitol dehydrogenase (XDH). Both XR and XDH preferentially utilize different coenzymes, NADPH and NAD^+^, respectively. Therefore, xylitol accumulates in yeast cells and is secreted into the fermentation medium due to the intracellular redox imbalance caused by the coenzyme preference, leading to poor ethanol yield (Chu and Lee 2007; van Vleet and Jeffries 2009; Matsushika et al. 2009). Another xylose assimilation pathway is catalyzed by xylose isomerase (XI), which directly converts xylose to xylulose. Therefore, XI-expressing strains do not produce excess xylitol. The isomerase pathway is suitable for high-yield ethanol production from xylose; however, the xylose consumption rate and ethanol productivity are low in the XI-expressing strains described thus far (van Maris et al. 2007; Matsushika et al. 2009). *S. cerevisiae* produces an endogenous non-specific aldose reductase encoded by *GRE3*. Deletion of *GRE3* decreases xylitol excretion not only in strains expressing XR and XDH but also in those expressing XI (Matsushika et al. 2009).

Overexpression of downstream metabolic genes such as *XKS1* and genes involved in the pentose phosphate pathway (PPP) has been used as a strategy to improve the xylose assimilation capacity of *S. cerevisiae* (Karhumaa et al. 2005; Kuyper et al. 2005a). As *S. cerevisiae* has no specific transporter for xylose, genes encoding heterologous and endogenous transporters with an affinity for xylose have also been overexpressed to improve xylose uptake (Matsushika et al. 2009). Evolutionary engineering has also been explored as a means to improve xylose utilization under aerobic and oxygen-limited conditions (Kuyper et al. 2005b; Zhou et al. 2012; Lee et al. 2014).

Disruption of *PHO13* by transposon mutagenesis was shown to improve xylose assimilation in a recombinant *S. cerevisiae* strain harboring the XR and XDH genes (Ni et al. 2007). Although PHO13p exhibits alkaline phosphatase activity, it remains unclear whether the protein functions in vivo. XR/XDH-based *PHO13* disruptants have demonstrated high ethanol productivity from xylose (van Vleet et al. 2008; Fujitomi et al. 2012; Shen et al. 2012; Kim et al. 2013; Li et al. 2014). Deletion of *PHO13* in an XI-expressing strain was also shown to effectively enhance cell growth from xylose (Lee et al. 2014), although the mechanism of the *PHO13*-deletion effect remains unclear.

In this study, *PHO13* was disrupted and *GRE3* deleted in a yeast strain that harbors multiple copies of the XI gene from *Orpinomyces* sp. and overexpresses the gene encoding XK. The effect of *PHO13* disruption on the metabolome was investigated by both transcriptome and metabolite analysis.

## Materials and methods

### Strains and media

*Escherichia coli* NovaBlue (Novagen, Inc., Madison, WI, USA) was used as the host strain for recombinant DNA manipulation and was grown in Luria–Bertani medium (10 g/L tryptone, 5 g/L yeast extract, and 5 g/L sodium chloride) containing 100 mg/L ampicillin. *S. cerevisiae* YPH499 (*MAT*a *ade2 his3 leu2 lys2 trp1 ura3*, purchased from Stratagene, La Jolla, CA, USA) was used as the host strain. Yeast strains were routinely cultivated at 30 °C in synthetic dextrose (SD) medium [6.7 g/L yeast nitrogen base without amino acids (Difco Laboratories, Detroit, MI, USA) and 20 g/L glucose] supplemented with appropriate amino and nucleic acids.

### Construction of plasmids

A DNA fragment containing the *S. cerevisiae* XK gene (*XKS1*) was amplified from plasmid pRS406XKS (Madhavan et al. 2009a) as a template using the primer set TDH3-XK F (5′-CCGCACCAGTTCTCACACGGAACACCACTA-3′) and TDH3-XK R (5′-CCACCGCGGTCAATCAATGAATCGAAAATG-3′). The amplified fragment was digested with *Sac*II and then ligated into the *Nae*I-*Sac*II site of pRS406 to yield plasmid pIUXK. A DNA fragment containing the *Orpinomyces* sp. XI gene (*xylA*) (Madhavan et al. 2009a, b) was amplified using the primer set XI F (5′-ATTGAATTCATGACTAAGGAATATTTCCC-3′) and XI R (5′-AATGTCGACTTATTGGTACATGGCAACAA-3′). After digestion with *Eco*RI and *Sal*I, the *xylA*-containing fragment was ligated into the *Eco*RI-*Sal*I site of pGK423 (Ishii et al. 2009). The fragment containing the *PGK1* promoter, *xylA*, and the *PGK1* terminator was digested with *Not*I and *Xho*I and ligated into the *Not*I-*Xho*I site of the chromosomal integration plasmid pδW (Yamada et al. 2010) to yield plasmid pδWXI.

### Yeast transformation

A *GRE3*-knockout strain of YPH499 (YΔG) was constructed using the PCR-mediated seamless gene deletion and marker recycling method (Akada et al. 2006). Plasmid pIUXK, digested with *Eco*RV, was transformed into YΔG to yield the XK-overexpressing strain YΔG/XK. Plasmid pδWXI, digested with *Asc*I, was transformed into YΔG/XK to yield strain YΔG/XK/XI. Transformants obtained using the lithium-acetate method (Chen et al. 1992) were selected on SD medium supplemented with appropriate amino acids and nucleotides. For the disruption of *PHO13* in YΔG/XK/XI, a DNA fragment that included *KanMX* was amplified from *S. cerevisiae* BY4741Δ*PHO13* (Invitrogen, Carlsbad, CA, USA) genomic DNA as a template using the primer set dPHO13-KanMX F (5′-CAAAAAAAGCCTTATAGCTTGCCCTGACAAAGAATATACAACTCGGGAAAAGATCTGTTTAGCTTGCCTCGTCCC-3′) and dPHO13-KanMX R (5′-ATTTTTCCTTTTCAAAAAGTAATTCTACCCCTAGATTTTGCATTGCTCCTGAGCTCGTTTTCGACACTGGATGGC-3′). The DNA fragment was transformed into YΔG/XK/XI to yield YΔGP/XK/XI. YΔGP/XK/XI was isolated on YPD (20 g/L peptone, 10 g/L yeast extract, 20 g/L glucose) plates containing 500 μg/L G418.

### XI activity assay

Extracts of yeast cells for use in the XI activity assay were prepared according to a previously reported method (Zhou et al. 2012). *S. cerevisiae* was cultivated in SD medium for 24 h, and cells were collected by centrifugation for 5 min at 2300×*g* and 4 °C. The pellet was washed twice with chilled washing buffer (10 mM phosphate buffer, 2 mM EDTA, pH 7.5) and suspended in chilled extraction buffer (100 mM phosphate buffer, 2 mM MgCl_2_, 1 mM dithiothreitol, pH 7.5). The suspended cells were mixed with glass beads (0.5-mm diameter), disrupted by shaking at 1500 rpm for 5 min using a Shake Master Neo (Biomedical Science, Tokyo, Japan), and centrifuged at 21,000×*g* and 4 °C for 15 min. The supernatant was collected as the cell extract and mixed with an assay mixture containing 100 mM Tris–HCl buffer (pH 7.5), 10 mM MgCl_2_, 0.15 mM NADH, and 2 U/mL sorbitol dehydrogenase. The assay was performed at 30 °C for 5 min. A 340-nm extinction coefficient for NADH of 6.3 mM^−1^ cm^−1^ was used to calculate the specific activity of XI. One unit of XI activity was defined as the amount of enzyme required to produce 1 μmol of xylulose per min under the assay conditions.

### Determination of the *xylA* copy number

Genomic DNA from YΔG/XK/XI and YΔGP/XK/XI was extracted using a Dr. GenTLE (yeast) high-recovery kit (Takara Bio, Shiga, Japan). The copy number of the *xylA* gene integrated into the chromosomes of the recombinant strains was determined using quantitative real-time PCR (qRT-PCR) performed on an Mx3000P QPCR System (Agilent Technologies, Palo Alto, CA, USA) with Thunderbird SYBR qPCR Mix (Toyobo, Osaka, Japan). The copy number was calculated according to the standard curve method (De Preter et al. 2002; Jin et al. 2003), with *PGK1* serving as the housekeeping gene. The following PCR primers were used to detect *PGK1* and *xylA*, respectively: qXI F, 5′-GATGCTGGTATGCTCGGTTCTA-3′ and qXI R, 5′-AACCTCCACCACGGATGATT-3′; qPGK1 F, 5′-CTTCGGTACCGCTCACAGAG-3′ and qPGK1 R, 5′-CTTGTCAGCAACCTTGGCAC-3′.

### Batch fermentation

After pre-incubation in 5 mL of SD medium for 24 h at 30 °C, YΔG/XK/XI and YΔGP/XK/XI cells were cultivated in 500 mL of YPD medium for 48 h at 30 °C under aerobic conditions. Yeast cells were collected by centrifugation at 2500×*g* for 10 min at 4 °C and then washed twice with distilled water. The cells were inoculated into 50 mL of YP medium (10 g/L yeast extract and 20 g/L peptone) containing 50 g/L xylose or 50 g/L glucose. The initial cell density for fermentation was set at 50 g wet cells/L. Ethanol fermentation was carried out at 30 °C with a mild agitation in 100-mL closed bottles equipped with a bubbling CO_2_ outlet. Substrate and product concentrations were determined by high-performance liquid chromatography (HPLC) on a Shimadzu HPLC system (Kyoto, Japan) equipped with a Shim-pack SPR-Pb column (7.8 mm × 250 mm, particle size 8 μm; Shimadzu) and an RID-10A refractive index detector (Shimadzu). The HPLC system was operated at 80 °C, with water as the mobile phase at a flow rate of 0.6 mL/min.

### Metabolite analysis by liquid chromatography–triple quadrupole mass spectrometry

Intracellular metabolites were prepared by leakage-free quenching and cold methanol extraction (Hasunuma et al. 2011). Liquid chromatography–triple quadrupole mass spectrometry (LC-QqQ-MS) was performed using (+)-10-camphorsulfonic acid as an internal standard. Metabolite extracts were evaporated under vacuum using a Free Zone 2.5 Plus system (Labconco, Kansas City, MO, USA), and the dried residues were stored at −80 °C until further use. Dried metabolites were dissolved in 50 μL of H_2_O before LC-QqQ-MS analysis. The LC-QqQ-MS system (LC: Agilent 1200 series; MS, Agilent 6460 with Jet Stream Technology; Agilent Technologies) was controlled using MassHunter Workstation Data Acquisition software (Agilent Technologies), as described previously (Kato et al. 2012). The following conditions were used for LC-QqQ-MS analyses: LC conditions, column, Mastro C18 (Shimadzu GLC Ltd.; 150 × 2.0 mm, particle size, 3 μm); mobile phase, 10 mM tributylamine and 15 mM acetic acid in H_2_O (A) and methanol (B); flow rate, 0.3 mL/min; gradient curve, 0 % B at 0 min, 0 % B at 8 min, 90 % B at 24 min, 0 % B at 24.1 min, and 0 % B at 30 min; injection volume, 5 μL; column temperature, 35 °C; mass analysis, negative ion mode; nebulizer flow, 55 psi; dry gas flow rate, 10 L/min at 300 °C; sheath gas flow rate, 11 L/min at 380 °C; capillary voltage, 3.5 kV; nozzle voltage, 1.0 kV; and detector voltage, 1.91 kV. Target metabolites were identified by comparing the chromatographic characteristics of sample peaks with those of authentic standards, and peak area was determined using MassHunter Quantitative Analysis ver. B04.00 software.

### DNA microarray analysis

Total RNA was obtained after 9 h of fermentation using a Total RNA Isolation Mini Kit (Agilent Technologies) according to the manufacturer’s protocol. The concentration and quality of RNA were determined using a NanoDrop ND-1000 spectrophotometer (NanoDrop, Wilmington, DE) and an Agilent 2100 Bioanalyzer (Agilent Technologies), respectively. cDNA was reverse transcribed and labeled with cyanine 3-CTP using a Low-Input Quick Amp Labeling Kit (Agilent Technologies) for hybridization to *S. cerevisiae* 4 × 44 k microarrays (Agilent Technologies). Hybridization was performed at 65 °C for 17 h. The arrays were scanned using an Agilent Single-Color DNA Microarray Scanner (Agilent Technologies). Gene expression levels were normalized per chip. Scan data were analyzed using GeneSpring GX ver. 11.5.1 software (Agilent Technologies). The experiment was performed in triplicate. The accession number of the microarray data was GSE73814.

## Results

### Construction of xylose-assimilating strains

A laboratory *S. cerevisiae* strain, YPH499, was used as the host for genetic engineering. The aldose reductase gene *GRE3*, which converts xylose to xylitol (Johansson et al. 2001), was deleted. Endogenous *XKS1*, encoding xylulokinase, was overexpressed under control of the *TDH3* promoter. This strain was referred to as YΔG/XK. The XI gene *xylA* from *Orpinomyces* sp. (Madhavan et al. 2009a, b) was integrated into the genome of the YΔG/XK. Previous studies reported that the rate of XI-catalyzed conversion of xylose to xylulose is very low in recombinant *S. cerevisiae* strains (van Maris et al. 2007; Matsushika et al. 2009). To increase the activity of XI, multiple copies of *xylA* were integrated into the δ sequence of YΔG/XK to yield YΔG/XK/XI. In YΔG/XK/XI, *PHO13* was disrupted to yield YΔGP/XK/XI.

### Determination of XI gene copy number and enzymatic activity

The *xylA* copy number was determined using qRT-PCR. The *xylA* copy number of YΔG/XI/XK (15.4 ± 0.7) was the same as that of YΔGP/XI/XK (15.0 ± 0.7). The enzyme activity of XI was 0.98 ± 0.12 and 0.82 ± 0.03 U/mg in YΔG/XI/XK and YΔGP/XI/XK, respectively.

### Glucose and xylose fermentation by recombinant strains

The effect of *PHO13* disruption on fermentation by the recombinant *S. cerevisiae* strains YΔG/XK/XI and YΔGP/XK/XI was investigated. After cultivation in YPD medium under aerobic conditions, yeast cells were transferred to YP medium containing 50 g/L glucose or 50 g/L xylose as the sole carbon source to initiate fermentation under oxygen-limited conditions.

**Fig. 1:**
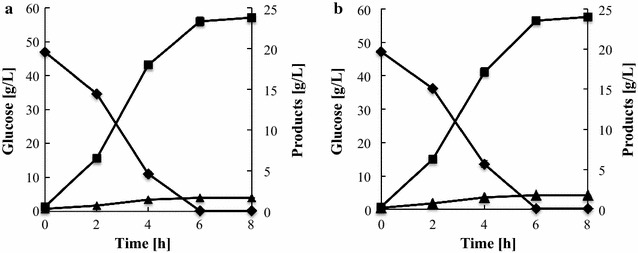
Oxygen-limited fermentation of glucose by **a** YΔG/XK/XI and **b** YΔGP/XK/XI. Glucose (*diamonds*), ethanol (*squares*), glycerol (*triangles*). Data are averages from three independent experiments

**Fig. 2:**
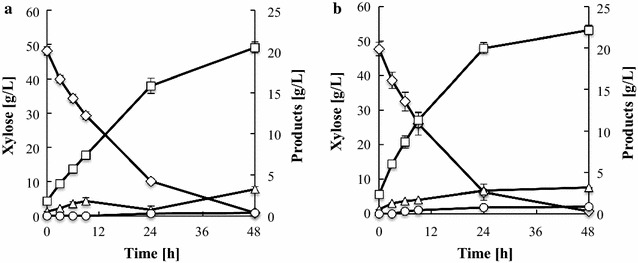
Oxygen-limited fermentation of xylose by **a** YΔG/XK/XI and **b** YΔGP/XK/XI. Xylose (*diamonds*), ethanol (*squares*), glycerol (*triangles*), xylitol (*circles*). Data are averages from three independent experiments

**Fig. 3:**
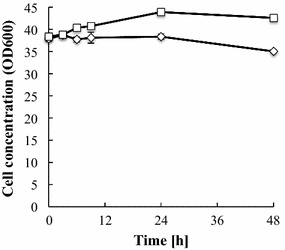
Cell growth during oxygen-limited fermentation. YΔG/XK/XI (*diamonds*) and YΔGP/XK/XI (*squares*). Data are averages from three independent experiments

During glucose fermentation, there was no difference in the glucose consumption rate or volumetric ethanol productivity between the two strains (Fig. 1). In contrast, during xylose fermentation, YΔGP/XK/XI demonstrated a higher xylose consumption rate (2.08 g/L/h) than YΔG/XK/XI (1.76 g/L/h) (Fig. 2; Table 1). Furthermore, the volumetric ethanol productivity of YΔGP/XK/XI was 1.5-fold higher than that of YΔG/XK/XI. The ethanol yield to theoretical carbon recovery yield (conversion ratio of 51.14 g ethanol from 100 g sugars was defined theoretically as 100 % carbon recovery yield) was 80.3 and 86.8 % in YΔG/XK/XI and YΔGP/XK/XI, respectively. These data indicate that disruption of *PHO13* has a positive impact on ethanol production from xylose in the XI-based strain. The concentration of YΔG/XK/XI cells remained nearly constant during xylose fermentation, whereas the density of YΔGP/XK/XI cells increased significantly (Fig. 3).

**Table 1:** Comparison of xylose fermentation performance of strains YΔG/XK/XI and YΔGP/XK/XI

Strain	Xylose consumption rate (g/L/h)	Volumetric ethanol productivity (g/L/h)	Ethanol yield (%)	Yield on total sugar		
				Ethanol (g/g-xylose)	Glycerol (g/g-xylose)	Xylitol (g/g-xylose)
YΔG/XK/XI	1.76 ± 0.01	0.57 ± 0.01	80.27 ± 2.59	0.42 ± 0.01	0.07 ± 0.01	0.01 ± 0.00
YΔGP/XK/XI	2.08 ± 0.21	0.88 ± 0.09	86.84 ± 2.21	0.45 ± 0.02	0.06 ± 0.00	0.02 ± 0.00

### DNA microarray transcriptome analysis during xylose fermentation

DNA microarray analysis was carried out to investigate the effect of *PHO13* disruption on global gene expression. RNA was extracted after 9 h of fermentation. The expression of the PPP-associated genes *GND1*, *SOL3*, *TAL1*, *RKI1*, *TKL1*, and *STB5* was upregulated following *PHO13* disruption. With respect to genes involved in glycolysis and gluconeogenesis, the expression of *ENO1* was upregulated and that of *GCR1*, *PYK2*, *SIP4*, and *RDS2* was downregulated. Upregulated expression of the TCA cycle and respiratory chain genes *NDE1*, *ACO1*, *ACO2*, *SDH2*, *IDH1*, *IDH2*, *ATP7*, *ATP19*, *SDH4*, *SDH3*, *CMC2*, and *ATP15* and downregulation of *PYC1* and *NDE2* were demonstrated. In ethanol and acetate biosynthesis from pyruvate, the expression of *ALD6* and *PDC1* was upregulated, while that of *ADH2*, *ALD5*, and *PDC6* was downregulated (Table 2). Interestingly, the expression of 125 genes involved in the cell cycle was altered by disruption of *PHO13* (Additional file 1: Tables S1, S2).

**Table 2:** Changes in gene expression after 9 h of oxygen-limited fermentation of xylose

Gene	Category	Fold change	Annotated function
*SOL3*	Pentose phosphate pathway	*3.96*	6-Phosphogluconolactonase
*GND1*		*3.60*	6-Phosphogluconatedehydrogenase
*TKL1*		*2.28*	Transketolase
*STB5*		*1.97*	Transcription factor
*TAL1*		*1.87*	Transaldolase
*RKI1*		*1.44*	Ribose-5-phosphate ketol-isomerase
*ENO1*	Glycolysis and gluconeogenesis	*1.26*	Phosphopyruvate hydratase
*RDS2*		1.22	Transcription factor
*GCR1*		1.23	Transcriptional activator
*PYK2*		1.43	Pyruvate kinase
*SIP4*		2.05	C6 zinc cluster transcriptional activator
*NDE1*	Tricarboxylic acid cycle and respiratory chain	*2.06*	Mitochondrial external NADH dehydrogenase, a type II NAD(P)H
*ACO1*		*2.03*	Aconitase
*ACO2*		*1.58*	Putative mitochondrial aconitase isozyme
*SDH2*		*1.52*	Iron-sulfur protein subunit of succinate dehydrogenase
*IDH1*		*1.40*	Subunit of mitochondrial NAD^+^-dependent isocitrate dehydrogenase
*IDH2*		*1.40*	Subunit of mitochondrial NAD^+^-dependent isocitrate dehydrogenase
*ATP7*		*1.32*	Subunit d of the stator stalk of mitochondrial F1F0 ATP synthase
*ATP19*		*1.30*	Subunit k of the mitochondrial F1F0 ATP synthase
*SDH4*		*1.25*	Membrane anchor subunit of succinate dehydrogenase
*SDH3*		*1.24*	Cytochrome b subunit of succinate dehydrogenase
*CMC2*		*1.23*	Protein of the mitochondrial intermembrane space with a role in respiratory chain complex assembly or maintenance
*ATP15*		*1.22*	Epsilon subunit of the F1 sector of mitochondrial F1F0 ATP synthase
*PYC1*		1.45	Pyruvate carboxylase isoform
*NDE2*		1.60	Mitochondrial external NADH dehydrogenase, catalyzes the oxidation of cytosolic NADH
*ALD6*	Ethanol production and utilization	*2.51*	Cytosolic aldehyde dehydrogenase
*PDC1*		*1.34*	Major of three pyruvate decarboxylase isozymes
*ALD5*		1.24	Mitochondrial aldehyde dehydrogenase
*PDC6*		1.87	Minor isoform of pyruvate decarboxylase
*ADH2*		3.20	Glucose-repressible alcohol dehydrogenase II

Fold change (*P* < 0.05) is the ratio of expression in YΔGP/XK/XI relative to that in YΔG/XK/XI. Italics indicates up-regulated expression in YΔGP/XK/XI

### Metabolite analysis using LC-QqQ-MS

**Fig. 4:**
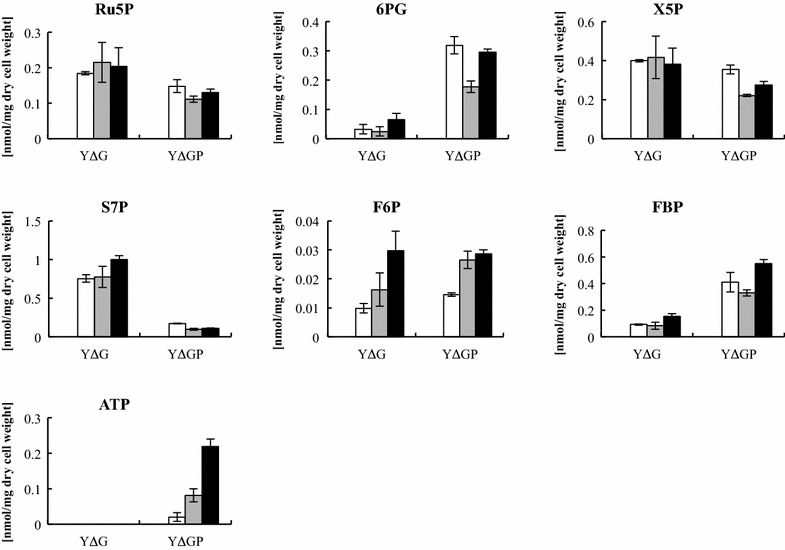
Intracellular metabolite concentrations in YΔG/XK/XI (YΔG) and YΔGP/XK/XI (YΔGP) after 3 h (*white bars*), 6 h (*gray bars*), and 9 h (*black bars*) of xylose fermentation. Data are averages from three independent experiments. *FBP* fructose-1,6-bisphosphate, *F6P* fructose-6-phosphate, *6PG* 6-phosphogluconate, *Ru5P* ribulose-5-phosphate, *S7P* sedoheptulose-7-phosphate, *X5P* xylulose-5-phosphate

Intracellular metabolites were extracted at 3, 6, and 9 h after the initiation of xylose fermentation. YΔGP/XK/XI demonstrated a significantly lower sedoheptulose 7-phosphate (S7P) level than YΔG/XK/XI (Fig. 4). The level of 6-phospho-d-gluconate (6PG) was higher in YΔGP/XK/XI than YΔG/XK/XI. The ATP level was increased in YΔGP/XK/XI.

## Discussion

In this study, xylose assimilation by *S. cerevisiae* was improved by integrating multiple copies of *xylA* (n = 15) into the yeast chromosome using the δ-integration method in combination with disruption of the *PHO13* gene. YΔGP/XK/XI demonstrated a xylose consumption rate of 2.08, 0.88 g/L/h volumetric ethanol productivity, and 86.8 % theoretical ethanol yield (0.45 g-ethanol/g-xylose), whereas the xylose consumption rate, volumetric ethanol productivity, and ethanol yield of the control strain YΔG/XK/XI were 1.76, 0.57 g/L/h, and 80.3 %, respectively (Table 1).

Recently, many metabolic engineering strategies have been employed to improve xylose assimilation by XI-expressing strains (Table 3). For example, Lee et al. (2014) constructed a strain designated SXA-R2P-E, which harbored two copies of the *PiromycesxylA* variant and overexpressed *Scheffersomyces stipitistal1* and endogenous *XKS1* in combination with disruption of *GRE3* and *PHO13*. Adaptive evolution of the SXA-R2P-E strain led to a xylose consumption rate of 0.98 and 0.44 g/g-cell/h specific ethanol productivity in anaerobic fermentation using a bioreactor (Table 3). Zhou et al. (2012) constructed a strain designated H131-A3-AL^CS^, which harbored multiple copies of *PiromycesxylA* and overexpressed *S. stipitisXYL3* and PPP genes (*S. stipitisTAL1* and endogenous *TKL1*, *RPE1*, and *RKI1*) in combination with the introduction of *ARG4* and *LEU2* for recovery of auxotrophic markers. Adaptive evolution of strain H131-A3-AL^CS^ led to a xylose consumption rate of 1.87 and 0.77 g/g-cell/h specific ethanol productivity in anaerobic fermentation using a bioreactor (Table 3). A number of XI-expressing, xylose-assimilating strains have been constructed, as noted above; however, these strains were constructed by complex gene recombination and adaptive evolution, which can be time-consuming and laborious. Adaptive evolution is widely used for improving xylose assimilation, although it is difficult to reproduce the same strain. In this study, we developed a novel xylose-assimilating strain, YΔGP/XK/XI, by combining δ-integration and *PHO13* disruption. YΔGP/XK/XI exhibited a xylose consumption rate of 0.31 and 0.11 g/g-cell/h specific ethanol productivity in oxygen-limited batch fermentation (Table 3).

**Table 3:** Comparison of xylose assimilation by *XI*-harboring recombinant strains

Strain	Description	Adaptation process	Fermentation conditions	Medium	Specific xylose consumption rate (g/g-cell/h)	Specific ethanol productivity (g/g-cell/h)	Ethanol yield (g/g-xylose)	References
YΔG/XK/XI	*Orpinimyces* *xylA*, *XKS1*, Δ*GRE3*	–	Oxygen-limited batch fermentation in 100-mL bottle	C	0.23	0.08	0.42	This study
YΔGP/XK/XI	*Orpinimyces* *xylA*, *XKS1*, Δ*GRE3*, Δ*PHO13*	–	Oxygen-limited batch fermentation in 100-mL bottle	C	0.31	0.11	0.45	This study
SXA-R2P-E	*Piromyces* *xylA* variant, *Scheffersomyces stipitis* *tal1*, *XKS1*, Δ*GRE3*, Δ*PHO13*, Evolved	Xylose adaptation	Oxygen-limited batch fermentation in 50-mL vial	S	0.14	0.05	NR	Lee et al. (2014)
SXA-R2P-E	*Piromyces* *xylA* variant, *Scheffersomyces stipitis* *tal1*, *XKS1*, Δ*GRE3*, Δ*PHO13*, Evolved	Xylose adaptation	Anaerobic batch fermentation in bioreactor	S + a. g.	0.98	0.44	0.45	Lee et al. (2014)
H131-A3-AL^CS^	*Piromyces* *xylA*, *S. stipitis XYL3*, *S. stipitis TAL1*, *TKL1*, *RPE1*, *RKI1*, *ARG4*, *LEU2*, Evolved	Xylose adaptation	Anaerobic batch fermentation in bioreactor	2 × S + a. g.	1.87	0.77	0.41	Zhou et al. (2012)
ADAP8	*Orpinimyces* *xylA*, *XKS1*, *SUT1*, Evolved	Xylose adaptation	Oxygen-limited batch fermentation in 100-mL bottle	C	NR	0.038	0.48	Madhavan et al. (2009b)
TMB3066	*Piromyces xylA*, *XKS1*, *TAL1*, *TKL1*, *RKI1*, *RPE1*, Δ*GRE3*	–	Anaerobic batch fermentation in 25-mL serum flask	S + a. g.	0.05	0.02	0.43	Karhumaa et al. (2007)
RWB 217	*Piromyces xylA*, *XKS1*, *TAL1*, *TKL1*, *RKI1*, *RPE1*, Δ*GRE3*	–	Anaerobic batch fermentation in bioreactor	S + a. g.	1.06	NR	0.43	Kuyper et al. (2005a)

*NR* not reported, *C* complex medium (10 g/L yeast extract, 20 g/L peptone), *S* synthetic medium (6.7 g/L yeast nitrogen base, appropriate nucleotides and amino acids), *a. g.* anaerobic growth factors (0.01 g/L ergosterol, 0.4 g/L Tween 80)

The effect of *PHO13* disruption on intracellular metabolism of XR/XDH-based strains has been investigated. Kim et al. (2015) reported upregulated expression of PPP and fermentative metabolism (glycolysis and ethanol production) genes (*GND1*, *SOL3*, *TAL1*, *GPM1*, *ENO1*, *CDC19*, *ADH1*, and *ADH5*) in an XR/XDH-expressing strain. In contrast, the expression of various genes involved in respiratory metabolism (TCA cycle and ethanol utilization; *PYC1*, *CIT3*, *ALD4*, *ADH2*, and *ACS1*) was downregulated by *PHO13* disruption. The transcription factor STB5, a zinc-finger protein that upregulates the expression of PPP and NADPH-producing genes under conditions of oxidative stress and NADPH limitation (Larochelle et al. 2006; Hector et al. 2009), was upregulated by *PHO13* disruption. The deletion of *PHO13* in an XR/XDH-overexpressing strain was shown to improve xylose fermentation (Ni et al. 2007; van Vleet et al. 2008; Shen et al. 2012; Fujitomi et al. 2012; Kim et al. 2013; Li et al. 2014). Kim et al. (2013) constructed a *PHO13* knockout strain SR7 *pho13Δ*, which indicate high specific ethanol productivity of 0.25 g/g-cell/h in oxygen limited fermentation. Li et al. (2014) constructed a *PHO13* knockout industrial strain NAPX37/Δ*PHO13*, which also indicates high specific ethanol productivity of 1.67 g/g-cell/h in oxygen limited fermentation. The enhancement of xylose assimilation ability is possibly due to upregulation of the PPP and elimination of the redox imbalance caused by NAD^+^ reduction by XR and NADPH oxidation by XDH (Kim et al. 2015).

In the present study, the effect of *PHO13* disruption on intracellular metabolism of the XI-expressing strain was investigated by transcriptome and metabolome analysis using DNA microarray and LC-QqQ-MS methods, respectively. We revealed that the expression of 12 TCA cycle and respiratory chain genes (*NDE1*, *ACO1*, *ACO2*, *SDH2*, *IDH1*, *IDH2*, *ATP7*, *ATP19*, *SDH4*, *SDH3*, *CMC2*, and *ATP15*) was upregulated (Table 2). The expression of PPP genes (*GND1*, *SOL3*, *TAL1*, *RKI1*, *TKL1*, and *STB5*) was upregulated. 125 genes involved in the cell cycle were dramatically altered by *PHO13* disruption (Additional file 1: Tables S1, S2). Metabolome analysis demonstrated a decrease in the level of S7P, an increase in the level of 6PG, and accumulation of ATP by *PHO13* deletion during xylose fermentation (Fig. 4).

According to previous studies, *S. cerevisiae* exhibits lower flux of the non-oxidative PPP than *S. stipitis* (Fiaux et al. 2003; Kötter and Ciriacy 1993) Utilization of S7P would be a rate-limiting step in the non-oxidative PPP (Jin et al. 2005; Johansson and Hahn-Hägerdal 2002; Kötter and Ciriacy 1993). Jin et al. (2003) indicated that the low flux of the non-oxidative PPP is likely due to insufficient supply of ATP required in excess activity of XK reaction. In fact, ATP is not accumulated in YΔG/XK/XI strain in this study (Fig. 4). Overexpression of PPP genes has improved ethanol yield and productivity in recombinant xylose-fermenting yeast strains (Kuyper et al. 2005a; Lu and Jeffries 2007). In the present study, the non-oxidative PPP genes (*TKL1*, *TAL1*, *RKI1*) was upregulated by the deletion of *PHO13*, which should be the reason of the reduction in S7P pool size (Fig. 4). The oxidative PPP genes (*SOL3* and *GND1*) and *STB5* encoding transcription factor was also upregulated by *PHO13* deletion. Kim et al. (2015) indicated STB5 upregulated *GND1* expression in a pho13Δ mutant. When recombinant xylose-fermenting *S. cerevisiae* strains assimilate xylose, metabolites produced via the non-oxidative PPP can be converted to 6PG via gluconeogenesis and the oxidative-PPP (Grotkjær et al. 2005; Wasylenko and Stephanopoulos 2015). 6PG is the substrate of GND1p. Since GND1p requires NADP^+^ as a coenzyme, upregulation of *GND1* might cause redox imbalance and accumulate 6PG by depletion of NADP^+^. Since the oxidative PPP generates CO_2_, reduced flux of the oxidative PPP might increase ethanol yield from xylose consumed.

Previously, *PHO13* disruptants indicated high growth ability on xylose medium (Fujitomi et al. 2012; Kim et al. 2013, 2015; Lee et al. 2014; Li et al. 2014; van Vleet et al. 2008). In the present study, YΔGP/XK/XI demonstrated slightly higher cell concentration than the control strain on xylose fermentation (Fig. 3). We found that *PHO13* disruption upregulates PPP-gene expression in *XI*-harboling strain and that transcriptional level of 125 cell cycle genes was altered (Additional file 1: Tables S1, S2). NADPH supplied by PPP is required for biosynthetic processes and biomass yield (Blank et al. 2005). Thus, PHO13 should be one of the factors affecting yeast cell growth.

This study is the first to demonstrate that disruption of *PHO13* enhances the xylose consumption rate, ethanol productivity, ethanol yield, and cell growth in an XI-expressing, xylose-fermenting yeast strain under oxygen-limited conditions (Fig. 2).

## Additional file

10.1186/s13568-015-0175-7 Down-regulated cell cycle genes after 9 h on oxygen-limited fermentation of xylose (**Table S1**) and up-regulated cell cycle genes after 9 h on oxygen-limited fermentation of xylose (**Table S2**).
