# Supplementary material for: Disruption of PHO13 improves ethanol production via the xylose isomerase pathway
Source: AMB Express. 2016 Jan 14;6:4. doi: 10.1186/s13568-015-0175-7 (PMC4713403; doi:10.1186/s13568-015-0175-7)
Supplement: Supplementary file 1 — 10.1186/s13568-015-0175-7 Down-regulated cell cycle genes after 9 h on oxygen-limited fermentation of xylose (Table S1) and up-regulated cell cycle genes after 9 h on oxygen-limited fermentation of xylose (Table S2). [file 13568_2015_175_MOESM1_ESM.pdf]

Supplementary material

AMB Express

Title:

Disruption of *PHO13* improves ethanol production via xylose isomerase pathway

Authors:

Takahiro Bamba[a], Tomohisa Hasunuma[b] and Akihiko Kondo[a,c]

Author Affiliation:

[a]Department of Chemical Science and Engineering, Graduate School of Engineering, Kobe University, 1-1 Rokkodai, Nada, Kobe 657-8501, Japan

[b]Organization of Advanced Science and Technology, Kobe University, 1-1 Rokkodai, Nada, Kobe 657-8501, Japan

[c]Biomass Engineering Program, RIKEN, 1-7-22 Suehiro-cho, Tsurumi-ku, Yokohama, Kanagawa 230-0045, Japan

Corresponding author:

Akihiko Kondo

Department of Chemical Science and Engineering

Graduate School of Engineering, Kobe University

1-1 Rokkodai, Nada, Kobe 657-8501, Japan

Telephone: +81-78-803-6196; Fax: +81-78-803-6196

E-mail: akondo@kobe-u.ac.jp

Table S1 Down-regulated cell cycle genes after 9 h on oxygen-limited fermentation of xylose. Fold change ($p < 0.05$) is the ratio of expression in YΔGP/XK/XI relative to YΔG/XK/XI

Gene name	Fold change	Annotated function
TAH11	1.86	"DNA replication licensing factor, required for pre-replication complex assembly" [YJR046W]
BFA1	1.62	"Component of the GTPase-activating Bfa1p-Bub2p complex involved in multiple cell cycle checkpoint pathways that control exit from mitosis" [YJR053W]
CLB4	1.55	"B-type cyclin involved in cell cycle progression; activates Cdc28p to promote the G2/M transition; may be involved in DNA replication and spindle assembly; accumulates during S phase and G2, then targeted for ubiquitin-mediated degradation" [YLR210W]
DOC1	1.54	"Processivity factor required for the ubiquitination activity of the anaphase promoting complex (APC), mediates the activity of the APC by contributing to substrate recognition; involved in cyclin proteolysis; contains a conserved DOC1 homology domain" [YGL240W]
MET30	1.50	"F-box protein containing five copies of the WD40 motif, controls cell cycle function, sulfur metabolism, and methionine biosynthesis as part of the ubiquitin ligase complex; interacts with and regulates Met4p, localizes within the nucleus" [YIL046W]
CDC20	1.47	"Cell-cycle regulated activator of anaphase-promoting complex/cyclosome (APC/C), which is required for metaphase/anaphase transition; directs ubiquitination of mitotic cyclins, Pds1p, and other anaphase inhibitors; potential Cdc28p substrate" [YGL116W]
ZIP2	1.43	"Meiosis-specific protein involved in normal synaptonemal complex formation and pairing between homologous chromosomes during meiosis" [YGL249W]
SPR28	1.42	"Sporulation-specific homolog of the yeast CDC3/10/11/12 family of bud neck microfilament genes; meiotic septin expressed at high levels during meiotic divisions and ascospore formation" [YDR218C]
DPB2	1.42	"Second largest subunit of DNA polymerase II (DNA polymerase epsilon), required for normal yeast chromosomal replication; expression peaks at the G1/S phase boundary; potential Cdc28p substrate" [YPR175W]
MCM1	1.41	"Transcription factor involved in cell-type-specific transcription and pheromone response; plays a central role in the formation of both repressor and activator complexes" [YMR043W]
YOR019W	1.37	"Protein of unknown function that may interact with ribosomes, based on co-purification experiments" [YOR019W]
YNG2	1.37	"Subunit of the NuA4 histone acetyltransferase complex that acetylates histone H4 and H2A; has similarity to the human tumor suppressor ING1 and its isoforms ING4 and ING5" [YHR090C]
PRP46	1.37	"Member of the NineTeen Complex (NTC) that contains Prp19p and stabilizes U6 snRNA in catalytic forms of the spliceosome containing U2, U5, and U6 snRNAs" [YPL151C]
SSP1	1.36	"Protein involved in the control of meiotic nuclear division and coordination of meiosis with spore formation; transcription is induced midway through meiosis" [YHR184W]
GID8	1.36	"Protein of unknown function, involved in proteasome-dependent catabolite inactivation of fructose-1,6-bisphosphatase; contains LisH and CTLH domains, like Vid30p; dosage-dependent regulator of START" [YMR135C]
MCM10	1.34	"Essential chromatin-associated protein involved in the initiation of DNA replication; required for the association of the MCM2-7 complex with replication origins" [YIL150C]
WHI3	1.31	"RNA binding protein that sequesters CLN3 mRNA in cytoplasmic foci; cytoplasmic retention factor for Cdc28p and associated cyclins; regulates cell fate and dose-dependently regulates the critical cell size required for passage through Start" [YNL197C]
MIH1	1.30	"Protein tyrosine phosphatase involved in cell cycle control; regulates the phosphorylation state of Cdc28p; homolog of S. pombe cdc25" [YMR036C]

SLG1	1.28	"Sensor-transducer of the stress-activated PKC1-MPK1 kinase pathway; involved in maintenance of cell wall integrity; required for mitophagy; involved in organization of the actin cytoskeleton; secretory pathway Wsc1p is required for the arrest of secretion response" [YOR008C]
INN1	1.25	"Essential protein that associates with the contractile actomyosin ring, required for ingression of the plasma membrane into the bud neck during cytokinesis; C2 domain, a membrane targeting module, is required for function" [YNL152W]
LDB19	1.24	"Protein involved in regulating the endocytosis of plasma membrane proteins by recruiting the ubiquitin ligase Rsp5p to its target; localization changes in response to nutrient levels; null mutant has reduced affinity for alcian blue dye" [YOR322C]
YPR109W	1.24	"Predicted membrane protein; dipoid deletion strain has high budding index" [YPR109W]
ELM1	1.22	"Serine/threonine protein kinase that regulates cellular morphogenesis, septin behavior, and cytokinesis; required for the regulation of other kinases; forms part of the bud neck ring" [YKL048C]
CDC7	1.22	"DDK (Dbf4-dependent kinase) catalytic subunit required for firing origins and replication fork progression in S phase through phosphorylation of Mcm2-7p complexes and Cdc45p; kinase activity correlates with cyclical DBF4 expression" [YDL017W]
BUD16	1.22	"Putative pyridoxal kinase, a key enzyme involved in pyridoxal 5'-phosphate synthesis, the active form of vitamin B6; required for genome integrity; involved in bud-site selection; similarity to yeast BUD17 and human pyridoxal kinase (PDXK)" [YEL029C]
CNN1	1.20	"Kinetochore protein of unknown function; associated with the essential kinetochore proteins Nnf1p and Spc24p; phosphorylated by both Clb5-Cdk1 and, to a lesser extent, Clb2-Cdk1." [YFR046C]
CEF1	1.20	"Essential splicing factor; associated with Prp19p and the spliceosome, contains an N-terminal c-Myb DNA binding motif necessary for cell viability but not for Prp19p association, evolutionarily conserved and homologous to S. pombe Cdc5p" [YMR213W]
RFC3	1.19	"Subunit of heteropentameric Replication factor C (RF-C), which is a DNA binding protein and ATPase that acts as a clamp loader of the proliferating cell nuclear antigen (PCNA) processivity factor for DNA polymerases delta and epsilon" [YNL290W]
SCC4	1.19	"Subunit of cohesin loading factor (Scc2p-Scc4p), a complex required for the loading of cohesin complexes onto chromosomes; involved in establishing sister chromatid cohesion during double-strand break repair via phosphorylated histone H2AX" [YER147C]
MCM22	1.18	"Protein involved in minichromosome maintenance; component of the kinetochore; binds to centromeric DNA in a Ctf19p-dependent manner" [YJR135C]

Table S2 Up-regulated cell cycle genes after 9 h on oxygen-limited fermentation of xylose. Fold change ($p < 0.05$) is the ratio of expression in YΔGP/XK/XI relative to YΔG/XK/XI

Gene name	Fold change	Annotated function
CLN1	7.14	"G1 cyclin involved in regulation of the cell cycle; activates Cdc28p kinase to promote the G1 to S phase transition; late G1 specific expression depends on transcription factor complexes, MBF (Swi6p-Mbp1p) and SBF (Swi6p-Swi4p)" [YMR199W]
PCL1	6.72	"Cyclin, interacts with cyclin-dependent kinase Pho85p; member of the Pcl1,2-like subfamily, involved in the regulation of polarized growth and morphogenesis and progression through the cell cycle; localizes to sites of polarized cell growth" [YNL289W]
YOX1	5.89	"Homeodomain-containing transcriptional repressor, binds to Mcm1p and to early cell cycle boxes (ECBs) in the promoters of cell cycle-regulated genes expressed in M/G1 phase; expression is cell cycle-regulated; potential Cdc28p substrate" [YML027W]
CLB2	4.91	"B-type cyclin involved in cell cycle progression; activates Cdc28p to promote the transition from G2 to M phase; accumulates during G2 and M, then targeted via a destruction box motif for ubiquitin-mediated degradation by the proteasome" [YPR119W]
CLB1	4.72	"B-type cyclin involved in cell cycle progression; activates Cdc28p to promote the transition from G2 to M phase; accumulates during G2 and M, then targeted via a destruction box motif for ubiquitin-mediated degradation by the proteasome" [YGR108W]
YHP1	4.70	"One of two homeobox transcriptional repressors (see also Yox1p), that bind to Mcm1p and to early cell cycle box (ECB) elements of cell cycle regulated genes, thereby restricting ECB-mediated transcription to the M/G1 interval" [YDR451C]
CLN2	3.85	"G1 cyclin involved in regulation of the cell cycle; activates Cdc28p kinase to promote the G1 to S phase transition; late G1 specific expression depends on transcription factor complexes, MBF (Swi6p-Mbp1p) and SBF (Swi6p-Swi4p)" [YPL256C]
CLB6	3.28	"B-type cyclin involved in DNA replication during S phase; activates Cdc28p to promote initiation of DNA synthesis; functions in formation of mitotic spindles along with Clb3p and Clb4p; most abundant during late G1" [YGR109C]
MCD1	3.10	"Essential subunit of the cohesin complex required for sister chromatid cohesion in mitosis and meiosis; apoptosis induces cleavage and translocation of a C-terminal fragment to mitochondria; expression peaks in S phase" [YDL003W]
CDC5	2.56	"Polo-like kinase with multiple functions in mitosis and cytokinesis through substrate phosphorylation, also functions in adaptation to DNA damage during meiosis; has similarity to Xenopus Plx1 and S. pombe Plo1p; possible Cdc28p substrate" [YMR001C]
ALK1	2.54	"Protein kinase; accumulation and phosphorylation are periodic during the cell cycle; phosphorylated in response to DNA damage; contains characteristic motifs for degradation via the APC pathway; similar to Alk2p and to mammalian haspins" [YGL021W]
EGT2	2.51	"Glycosylphosphatidylinositol (GPI)-anchored cell wall endoglucanase required for proper cell separation after cytokinesis, expression is activated by Swi5p and tightly regulated in a cell cycle-dependent manner" [YNL327W]
BUD4	2.48	"Involved in bud-site selection and required for the axial budding pattern; localizes with septins to bud neck in mitosis and may constitute an axial landmark for next round of budding; required for the formation of a double septin ring, and generally for the organization of septin structures; potential Cdc28p substrate" [YJR092W]
CDC45	2.35	"DNA replication initiation factor; recruited to MCM pre-RC complexes at replication origins; promotes release of MCM from Mcm10p, recruits elongation machinery; mutants in human homolog may cause velocardiofacial and DiGeorge syndromes" [YLR103C]
KCC4	2.34	"Protein kinase of the bud neck involved in the septin checkpoint, associates with septin proteins, negatively regulates Swe1p by phosphorylation, shows

		structural homology to bud neck kinases Gin4p and Hsl1p" [YCL024W]
RAX2	2.18	"N-glycosylated protein involved in the maintenance of bud site selection during bipolar budding; localization requires Rax1p; RAX2 mRNA stability is regulated by Mpt5p" [YLR084C]
TOF1	2.15	"Subunit of a replication-pausing checkpoint complex (Tof1p-Mrc1p-Csm3p) that acts at the stalled replication fork to promote sister chromatid cohesion after DNA damage, facilitating gap repair of damaged DNA; interacts with the MCM helicase" [YNL273W]
SCC2	2.15	"Subunit of cohesin loading factor (Scc2p-Scc4p), a complex required for loading of cohesin complexes onto chromosomes; involved in establishing sister chromatid cohesion during DSB repair via histone H2AX; evolutionarily-conserved adherin" [YDR180W]
PSA1	2.15	"GDP-mannose pyrophosphorylase (mannose-1-phosphate guanyltransferase), synthesizes GDP-mannose from GTP and mannose-1-phosphate in cell wall biosynthesis; required for normal cell wall structure" [YDL055C]
SGO1	2.14	"Component of the spindle checkpoint, involved in sensing lack of tension on mitotic chromosomes; protects centromeric Rec8p at meiosis I; required for accurate chromosomal segregation at meiosis II and for mitotic chromosome stability" [YOR073W]
ACM1	2.11	"Pseudosubstrate inhibitor of the anaphase-promoting complex/cyclosome (APC/C), that suppresses APC/C [Cdh1]-mediated proteolysis of mitotic cyclins; associates with Cdh1
MMR1	2.10	"Phosphorylated protein of the mitochondrial outer membrane, localizes only to mitochondria of the bud; interacts with Myo2p to mediate mitochondrial distribution to buds; mRNA is targeted to the bud via the transport system involving She2p" [YLR190W]
CTF18	1.97	"Subunit of a complex with Ctf8p that shares some subunits with Replication Factor C and is required for sister chromatid cohesion; may have overlapping functions with Rad24p in the DNA damage replication checkpoint" [YMR078C]
FAR1	1.86	"Cyclin-dependent kinase inhibitor that mediates cell cycle arrest in response to pheromone; also forms a complex with Cdc24p, Ste4p, and Ste18p that may specify the direction of polarized growth during mating; potential Cdc28p substrate" [YJL157C]
STU1	1.86	"Component of the mitotic spindle that binds to interpolar microtubules via its association with beta-tubulin (Tub2p); required for interpolar microtubules to provide an outward force on the spindle poles" [YBL034C]
DUO1	1.81	"Essential subunit of the Dam1 complex (aka DASH complex), couples kinetochores to the force produced by MT depolymerization thereby aiding in chromosome segregation; is transferred to the kinetochore prior to mitosis" [YGL061C]
DBF2	1.78	"Ser/Thr kinase involved in transcription and stress response; functions as part of a network of genes in exit from mitosis; localization is cell cycle regulated; activated by Cdc15p during the exit from mitosis" [YGR092W]
NRM1	1.76	"Transcriptional co-repressor of MBF (MCB binding factor)-regulated gene expression; Nrm1p associates stably with promoters via MBF to repress transcription upon exit from G1 phase" [YNR009W]
CDC6	1.76	"Essential ATP-binding protein required for DNA replication, component of the pre-replicative complex (pre-RC) which requires ORC to associate with chromatin and is in turn required for Mcm2-7p DNA association; homologous to S. pombe Cdc18p" [YJL194W]
SMC1	1.74	"Subunit of the multiprotein cohesin complex, essential protein involved in chromosome segregation and in double-strand DNA break repair; SMC chromosomal ATPase family member, binds DNA with a preference for DNA with secondary structure" [YFL008W]
PCL2	1.71	"Cyclin, interacts with cyclin-dependent kinase Pho85p; member of the Pcl1,2-like subfamily, involved in the regulation of polarized growth and morphogenesis and progression through the cell cycle; localizes to sites of polarized cell growth" [YDL127W]
SUN4	1.69	"Cell wall protein related to glucanases, possibly involved in cell wall septation; member of the SUN family" [YNL066W]
KIP1	1.69	"Kinesin-related motor protein required for mitotic spindle assembly, chromosome segregation, and 2 micron plasmid partitioning; functionally redundant with Cin8p for chromosomal but not plasmid functions" [YBL063W]
LTE1	1.66	"Protein similar to GDP/GTP exchange factors but without detectable GEF activity; required for asymmetric localization of Bfa1p at daughter-directed spindle pole bodies and for mitotic exit at low temperatures" [YAL024C]

SWE1	1.66	"Protein kinase that regulates the G2/M transition by inhibition of Cdc28p kinase activity; localizes to the nucleus and to the daughter side of the mother-bud neck; homolog of S. pombe Wee1p; potential Cdc28p substrate" [YJL187C]
BUD3	1.65	"Protein involved in bud-site selection and required for axial budding pattern; localizes with septins to bud neck in mitosis and may constitute an axial landmark for next round of budding" [YCL014W]
MOB2	1.62	"Activator of Cbk1p kinase; component of the RAM signaling network that regulates cellular polarity and morphogenesis; activation of Cbk1p facilitates the Ace2p-dependent daughter cell-specific transcription of genes involved in cell separation; similar to Mob1p" [YFL034C-B]
AMN1	1.59	"Protein required for daughter cell separation, multiple mitotic checkpoints, and chromosome stability; contains 12 degenerate leucine-rich repeat motifs; expression is induced by the Mitotic Exit Network (MEN)" [YBR158W]
RAD53	1.59	"Protein kinase, required for cell-cycle arrest in response to DNA damage; activated by trans autophosphorylation when interacting with hyperphosphorylated Rad9p; also interacts with ARS1 and plays a role in initiation of DNA replication" [YPL153C]
BUD9	1.56	"Protein involved in bud-site selection; mutant has increased aneuploidy tolerance; diploid mutants display a unipolar budding pattern instead of the wild-type bipolar pattern, and bud at the distal pole" [YGR041W]
YCG1	1.55	"Subunit of the condensin complex; required for establishment and maintenance of chromosome condensation, chromosome segregation and chromatin binding of the condensin complex; required for clustering of tRNA genes at the nucleolus" [YDR325W]
MTW1	1.54	"Essential component of the MIND kinetochore complex (Mtw1p Including Nnf1p-Nsl1p-Dsn1p) which joins kinetochore subunits contacting DNA to those contacting microtubules; critical to kinetochore assembly" [YAL034W-A]
HOF1	1.53	"Bud neck-localized, SH3 domain-containing protein required for cytokinesis; regulates actomyosin ring dynamics and septin localization; interacts with the formins, Bni1p and Bnr1p, and with Cyk3p, Vrp1p, and Bni5p" [YMR032W]
NUD1	1.53	"Component of the spindle pole body outer plaque, required for exit from mitosis" [YOR373W]
PDS5	1.52	"Protein required for establishment and maintenance of sister chromatid condensation and cohesion, colocalizes with cohesin on chromosomes, may function as a protein-protein interaction scaffold; also required during meiosis" [YMR076C]
ELG1	1.52	"Subunit of an alternative replication factor C complex important for DNA replication and genome integrity; suppresses spontaneous DNA damage; involved in homologous recombination-mediated repair and telomere homeostasis" [YOR144C]
RSR1	1.51	"GTP-binding protein of the ras superfamily required for bud site selection, morphological changes in response to mating pheromone, and efficient cell fusion; localized to the plasma membrane; significantly similar to mammalian Rap GTPases" [YGR152C]
CIN8	1.49	"Kinesin motor protein involved in mitotic spindle assembly and chromosome segregation" [YEL061C]
PSF1	1.49	"Subunit of the GINS complex (Sld5p, Psf1p, Psf2p, Psf3p), which is localized to DNA replication origins and implicated in assembly of the DNA replication machinery" [YDR013W]
KAR1	1.49	"Essential protein involved in karyogamy during mating and in spindle pole body duplication during mitosis, localizes to the half-bridge of the spindle pole body, interacts with Spc72p during karyogamy, also interacts with Cdc31p" [YNL188W]
MCM7	1.49	"Component of the heterohexameric MCM2-7 complex, which primes origins of DNA replication in G1 and becomes an active ATP-dependent helicase that promotes DNA melting and elongation in S-phase; forms an Mcm4p-6p-7p subcomplex" [YBR202W]
CLN3	1.48	"G1 cyclin involved in cell cycle progression; activates Cdc28p kinase to promote the G1 to S phase transition; plays a role in regulating transcription of the other G1 cyclins, CLN1 and CLN2; regulated by phosphorylation and proteolysis" [YAL040C]
SMC2	1.47	"Subunit of the condensin complex; essential SMC chromosomal ATPase family member that forms a complex with Smc4p to form the active ATPase; Smc2p/Smc4p complex binds DNA; required for clustering of tRNA genes at the nucleolus" [YFR031C]
DBF4	1.46	"Regulatory subunit of Cdc7p-Dbf4p kinase complex, required for Cdc7p kinase activity and initiation of DNA replication; phosphorylates the Mcm2-7

		family of proteins; cell cycle regulated" [YDR052C]
BNI5	1.44	"Protein involved in organization of septins at the mother-bud neck, may interact directly with the Cdc11p septin, localizes to bud neck in a septin-dependent manner" [YNL166C]
DAD3	1.44	"Essential subunit of the Dam1 complex (aka DASH complex), couples kinetochores to the force produced by MT depolymerization thereby aiding in chromosome segregation; is transferred to the kinetochore prior to mitosis" [YBR233W-A]
IPL1	1.44	"Aurora kinase subunit of the conserved chromosomal passenger complex (CPC; Ipl1p-Sli15p-Bir1p-Nbl1p), involved in regulating kinetochore-microtubule attachments; helps maintain condensed chromosomes during anaphase and early telophase" [YPL209C]
SHE1	1.41	"Mitotic spindle protein that interacts with components of the Dam1 (DASH) complex, its effector Sli15p, and microtubule-associated protein Bim1p; also localizes to nuclear microtubules and to the bud neck in a ring-shaped structure" [YBL031W]
SLK19	1.38	"Kinetochore-associated protein required for normal segregation of chromosomes in meiosis and mitosis; component of the FEAR regulatory network, which promotes Cdc14p release from the nucleolus during anaphase; potential Cdc28p substrate" [YOR195W]
CDC10	1.37	"Component of the septin ring that is required for cytokinesis; septins recruit proteins to the mother-bud neck and can act as a barrier to diffusion at the membrane, and they comprise the 10 nm filaments seen with EM; required for the transition from a single to double septin ring" [YCR002C]
CDC3	1.34	"Component of the septin ring of the mother-bud neck that is required for cytokinesis; septins recruit proteins to the neck and can act as a barrier to diffusion at the membrane, and they comprise the 10nm filaments seen with EM" [YLR314C]
KAR3	1.34	"Minus-end-directed microtubule motor that functions in mitosis and meiosis, localizes to the spindle pole body and localization is dependent on functional Cik1p, required for nuclear fusion during mating; potential Cdc28p substrate" [YPR141C]
CDC14	1.34	"Protein phosphatase required for mitotic exit; located in the nucleolus until liberated by the FEAR and Mitotic Exit Network in anaphase, enabling it to act on key substrates to effect a decrease in CDK/B-cyclin activity and mitotic exit; required for meiosis I spindle disassembly; released from nucleolus upon entry into anaphase I of meiosis, resequestered in metaphase II, then released again upon entry into anaphase II; maintained in nucleolus by Cdc55p in early meiosis" [YFR028C]
MCM2	1.33	"Protein involved in DNA replication; component of the Mcm2-7 hexameric complex that binds chromatin as a part of the pre-replicative complex" [YBL023C]
PAP2	1.33	"Non-canonical poly(A) polymerase, involved in nuclear RNA degradation as a component of the TRAMP complex; catalyzes polyadenylation of hypomodified tRNAs, and snoRNA and rRNA precursors; overlapping but non-redundant functions with Trf5p" [YOL115W]
YCS4	1.33	"Subunit of the condensin complex; required for establishment and maintenance of chromosome condensation, chromosome segregation, chromatin binding of condensin, tRNA gene clustering at the nucleolus, and silencing at the mating type locus" [YLR272C]
NNF1	1.32	"Essential component of the MIND kinetochore complex (Mtw1p Including Nnf1p-Nsl1p-Dsn1p) which joins kinetochore subunits contacting DNA to those contacting microtubules; required for accurate chromosome segregation" [YJR112W]
RIF1	1.32	"Protein that binds to the Rap1p C-terminus and acts synergistically with Rif2p to help control telomere length and establish telomeric silencing; deletion results in telomere elongation" [YBR275C]
CLB5	1.32	"B-type cyclin involved in DNA replication during S phase; activates Cdc28p to promote initiation of DNA synthesis; functions in formation of mitotic spindles along with Clb3p and Clb4p; most abundant during late G1 phase" [YPR120C]
MMS22	1.32	"Protein that acts with Mms1p in a repair pathway that may be involved in resolving replication intermediates or preventing the damage caused by blocked replication forks; required for accurate meiotic chromosome segregation" [YLR320W]
TEM1	1.32	"GTP-binding protein of the ras superfamily involved in termination of M-phase; controls actomyosin and septin dynamics during cytokinesis" [YML064C]
DSE1	1.31	"Daughter cell-specific protein, may regulate cross-talk between the mating and filamentation pathways; deletion affects cell separation after division and sensitivity to alpha-factor and drugs affecting the cell wall" [YER124C]

SAP4	1.30	"Protein required for function of the Sit4p protein phosphatase, member of a family of similar proteins that form complexes with Sit4p, including Sap155p, Sap185p, and Sap190p" [YGL229C]
ECO1	1.30	"Acetyltransferase required for establishment of sister chromatid cohesion; modifies Smc3p at replication forks and Mcd1p in response to ds DNA breaks; has a C2H2-type zinc finger; mutations in human homolog ESCO2 cause Roberts syndrome" [YFR027W]
IQG1	1.30	"Essential protein required for determination of budding pattern, promotes localization of axial markers Bud4p and Cdc12p and functionally interacts with Sec3p, localizes to the contractile ring during anaphase, member of the IQGAP family" [YPL242C]
CHL1	1.29	"Probable DNA helicase involved in sister-chromatid cohesion and genome integrity; interacts with ECO1 and CTF18; mutants are defective in silencing, rDNA recombination, aging and the heat shock response; FANCJ-like helicase family member" [YPL008W]
DAD2	1.29	"Essential subunit of the Dam1 complex (aka DASH complex), couples kinetochores to the force produced by MT depolymerization thereby aiding in chromosome segregation; is transferred to the kinetochore prior to mitosis" [YKR083C]
CDC27	1.29	"Subunit of the Anaphase-Promoting Complex/Cyclosome (APC/C), which is a ubiquitin-protein ligase required for degradation of anaphase inhibitors, including mitotic cyclins, during the metaphase/anaphase transition" [YBL084C]
SLD2	1.28	"Single-stranded DNA origin-binding and annealing protein; required for the initiation of DNA replication; phosphorylated in S phase by cyclin-dependent kinases (Cdks), promoting origin binding, DNA replication and Dpb11p complex formation; component of the preloading complex; required for the S phase checkpoint" [YKL108W]
CYK3	1.28	"SH3-domain protein located in the mother-bud neck and the cytokinetic actin ring; mutant phenotype and genetic interactions suggest a role in cytokinesis" [YDL117W]
CKS1	1.27	"Cyclin-dependent protein kinase regulatory subunit and adaptor; modulates proteolysis of M-phase targets through interactions with the proteasome; role in transcriptional regulation, recruiting proteasomal subunits to target gene promoters" [YBR135W]
PDS1	1.27	"Securin, inhibits anaphase by binding separin Esp1p; blocks cyclin destruction and mitotic exit, essential for meiotic progression and mitotic cell cycle arrest; localization is cell-cycle dependent and regulated by Cdc28p phosphorylation" [YDR113C]
APC1	1.27	"Largest subunit of the Anaphase-Promoting Complex/Cyclosome (APC/C), which is a ubiquitin-protein ligase required for degradation of anaphase inhibitors, including mitotic cyclins, during the metaphase/anaphase transition; component of the platform domain of the APC/C, based on structural analysis" [YNL172W]
SDS22	1.26	"Conserved nuclear regulatory subunit of Glc7p type 1 protein serine-threonine phosphatase (PP1), functions positively with Glc7p to promote dephosphorylation of nuclear substrates required for chromosome transmission during mitosis" [YKL193C]
NOC3	1.26	"Protein that forms a nuclear complex with Noc2p that binds to 66S ribosomal precursors to mediate their intranuclear transport; also binds to chromatin to promote the association of DNA replication factors and replication initiation" [YLR002C]
DSN1	1.25	"Essential component of the MIND kinetochore complex (Mtw1p Including Nnf1p-Nsl1p-Dsn1p) which joins kinetochore subunits contacting DNA to those contacting microtubules; important for chromosome segregation" [YIR010W]
BUD8	1.25	"Protein involved in bud-site selection; diploid mutants display a unipolar budding pattern instead of the wild-type bipolar pattern, and bud at the proximal pole" [YLR353W]
RAD24	1.23	"Checkpoint protein, involved in the activation of the DNA damage and meiotic pachytene checkpoints; subunit of a clamp loader that loads Rad17p-Mec3p-Ddc1p onto DNA; homolog of human and S. pombe Rad17 protein" [YER173W]
SAP185	1.23	"Protein that forms a complex with the Sit4p protein phosphatase and is required for its function; member of a family of similar proteins including Sap4p, Sap155p, and Sap190p" [YJL098W]
DAD1	1.23	"Essential subunit of the Dam1 complex (aka DASH complex), couples kinetochores to the force produced by MT depolymerization thereby aiding in chromosome segregation; is transferred to the kinetochore prior to mitosis" [YDR016C]

PCL9	1.23	"Cyclin, forms a functional kinase complex with Pho85p cyclin-dependent kinase (Cdk), expressed in late M/early G1 phase, activated by Swi5p" [YDL179W]
MAD3	1.21	"Subunit of the spindle-assembly checkpoint complex, which delays anaphase onset in cells with defects in mitotic spindle assembly; pseudosubstrate inhibitor of APC(Cdc20), the anaphase promoting complex involved in securin (Pds1p) turnover" [YJL013C]
DPB11	1.21	"Replication initiation protein that loads DNA pol epsilon onto pre-replication complexes at origins; checkpoint sensor recruited to stalled replication forks by the checkpoint clamp complex where it activates Mec1p; ortholog of human TopBP1" [YJL090C]
CSM3	1.21	"Replication fork associated factor, required for stable replication fork pausing; component of the DNA replication checkpoint pathway; required for accurate chromosome segregation during meiosis" [YMR048W]
CDC28	1.20	"Catalytic subunit of the main cell cycle cyclin-dependent kinase (CDK); alternately associates with G1 cyclins (CLNs) and G2/M cyclins (CLBs) which direct the CDK to specific substrates" [YBR160W]
